# Elevated Atherosclerosis-Related Gene Expression, Monocyte Activation and Microparticle-Release Are Related to Increased Lipoprotein-Associated Oxidative Stress in Familial Hypercholesterolemia

**DOI:** 10.1371/journal.pone.0121516

**Published:** 2015-04-13

**Authors:** Morten Hjuler Nielsen, Helle Irvine, Simon Vedel, Bent Raungaard, Henning Beck-Nielsen, Aase Handberg

**Affiliations:** 1 Danish PhD School of Molecular Metabolism, University of Southern Denmark, Odense, Denmark; 2 Department of Medicine and Cardiology A, Aarhus University Hospital, Aarhus, Denmark; 3 Department of Radiology, Aarhus University Hospital, Aarhus, Denmark; 4 Department of Cardiology, Aalborg University Hospital, Aalborg, Denmark; 5 Department of Endocrinology M, Odense University Hospital, Odense, Denmark; 6 Department of Clinical Biochemistry, Aalborg University Hospital, Aalborg, Denmark; Centre d'Immunologie et des Maladies Infectieuses,INSERM, FRANCE

## Abstract

**Objective:**

Animal and in vitro studies have suggested that hypercholesterolemia and increased oxidative stress predisposes to monocyte activation and enhanced accumulation of oxidized LDL cholesterol (oxLDL-C) through a CD36-dependent mechanism. The aim of this study was to investigate the hypothesis that elevated oxLDL-C induce proinflammatory monocytes and increased release of monocyte-derived microparticles (MMPs), as well as up-regulation of CD36, chemokine receptors and proinflammatory factors through CD36-dependent pathways and that this is associated with accelerated atherosclerosis in subjects with heterozygous familial hypercholesterolemia (FH), in particular in the presence of Achilles tendon xanthomas (ATX).

**Approach and Results:**

We studied thirty FH subjects with and without ATX and twenty-three healthy control subjects. Intima-media thickness (IMT) and Achilles tendon (AT) thickness were measured by ultrasonography. Monocyte classification and MMP analysis were performed by flow cytometry. Monocyte expression of genes involved in atherosclerosis was determined by quantitative PCR. IMT and oxLDL-C were increased in FH subjects, especially in the presence of ATX. In addition, FH subjects had elevated proportions of intermediate CD14++CD16+ monocytes and higher circulating MMP levels. Stepwise linear regression identified oxLDL-C, gender and intermediate monocytes as predictors of MMPs. Monocyte expression of pro-atherogenic and pro-inflammatory genes regulated by oxLDL-C-CD36 interaction was increased in FH, especially in ATX+ subjects. Monocyte chemokine receptor CX_3_CR1 was identified as an independent contributor to IMT.

**Conclusions:**

Our data support that lipoprotein-associated oxidative stress is involved in accelerated atherosclerosis in FH, particularly in the presence of ATX, by inducing pro-inflammatory monocytes and increased release of MMPs along with elevated monocyte expression of oxLDL-C-induced atherosclerosis-related genes.

## Introduction

The attachment and subsequent transmigration of circulating monocytes into the subendothelial space is facilitated by hypercholesterolemia-induced expression of adhesion molecules on endothelial cells and their secretion of chemoattractant factors. The monocytes differentiate into macrophages, which internalize lipoproteins and become proinflammatory resulting in further recruitment of monocytes and promoting inflammation and progression of atherosclerosis as reviewed in [1]. Previous studies have indicated that circulating monocytes are a heterogenic population composed of at least two distinct subpopulations based on surface expressions of CD14 and CD16 [2]. The major subpopulation expresses high levels of CD14 and low levels of CD16, whereas the minor and more proinflammatory subpopulation expresses low levels of CD14 and high levels of CD16 on the cell surface [3]. A subset of CD16-positive monocytes produces high levels of inflammatory mediators and up-regulates a number of chemokine receptors, including CCR2, CX_3_CR1 and CCR5 which are attributed non-redundant and independent roles in the development of atherosclerosis [4,5].

Uptake of oxidized LDL cholesterol (oxLDL-C) by the scavenger receptor CD36 in monocytes and macrophages leads to an up-regulation of CD36 expression through activation of the transcription factor PPAR-γ, thereby creating a ‘vicious’ feed-forward cycle of increasing oxLDL-C uptake, ultimately converting the monocyte/macrophage into an atherogenic foam cell as reviewed in [6]. Other effects of oxLDL-C binding to CD36 include activation of the transcription factor NFκB which induces production of proinflammatory cytokines and a proinflammatory phenotype [7].

Familial hypercholesterolemia (FH) is an autosomal codominant genetic disorder of lipoprotein metabolism [8], characterized by elevated plasma levels of LDL cholesterol, a high incidence of premature coronary heart disease and extravascular deposits of cholesterol in tendons (tendon xanthomas) [9]. The presence of Achilles tendon xanthomas (ATX) is a marker for high risk of cardiovascular disease among FH patients [10,11] and xanthomas and atherosclerosis may result from the same pathophysiological mechanisms. Microparticles (MPs) are vesicles (< 1μm) shed from the plasma membranes of activated circulating and vascular cells and are considered to constitute a new inter-cellular signaling system which may be involved in various diseases such as cardiovascular disorders [12].

The overall purpose of the present study was to investigate the involvement of monocytes and lipoprotein-associated oxidative stress in the atherosclerotic process. Our hypothesis was that elevated oxLDL-C in FH induce proinflammatory monocytes and increased release of monocyte-derived microparticles (MMPs), as well as up-regulation of CD36, chemokine receptors and proinflammatory factors through CD36-dependent pathways, and that this accelerates atherosclerosis.

To study this, the proportion of CD16-positive monocyte subpopulations in peripheral blood of FH subjects with and without ATX was compared and CD36 surface expression levels determined. Monocyte expression levels of selected genes involved in the atherosclerotic process and known to be induced by oxLDL-C were determined and related to IMT. Furthermore, as atherosclerosis and Achilles tendon thickening may share common mechanisms we evaluated this relationship in FH. Finally, MMPs were quantified, and the association between proinflammatory monocyte subpopulations, oxLDL-C and circulating MMPs was studied.

## Materials and Methods

### Study population

The study group comprised thirty patients (18 females and 12 males) genetically diagnosed with heterozygous FH and selected on the basis of the presence or absence of ATX according to medical records. Twenty-three healthy controls (15 females and 8 males), as indicated by a medical questionnaire, served as the control group. Exclusion criteria were: Hypertension, diabetes mellitus, previous history of atherosclerotic disease and the use of lipid-lowering medication (except for FH subjects). FH patients were treated with lipid-lowering therapy at inclusion and underwent an 8-week washout period before blood sampling [13]. The study was conducted in agreement with the Helsinki-II declaration and approved by The Central Denmark Region Committees on Health Research (2010–0147) and by the Danish Data Protection Agency (j.no.: 2010-41-4879). Written informed consent was obtained from all participants. Blood pressure, height and body weight were recorded and blood samples were obtained in the fasting state. The following parameters were determined in the routine laboratory: Platelet, leukocyte and monocyte counts, as well as hemoglobin levels were determined on a Sysmex XE-5000 Automated Hematology System (EDTA plasma). A Cobas 6000 analyzer (Roche) was used to determine concentrations of glucose, alanine-aminotransferase (ALT), triglycerides, total cholesterol (Total-C), low-density lipoprotein cholesterol (LDL-C), high-density lipoprotein cholesterol (HCL-C) and apolipoprotein B (ApoB) (Li-Heparin plasma). LDL-C concentrations were estimated from the Friedewald formula [14].

### Markers of lipoprotein-associated oxidative stress and inflammation

Oxidized LDL-C (Mercodia, Uppsala, Sweden), C-Reactive Protein (CRP) (MBL International, Woburn, MA, USA), Interleukin-6 (IL-6) (Diaclone, Besancon Cedex, France), Matrix Metalloproteinase 9 (MMP-9) (Nordic BioSite, Denmark) and Intercellular Adhesion Molecule 1 (ICAM-1) (R&D Systems, Europe) were measured on EDTA plasma. All ELISA measurements were performed according to the manufacturer’s instructions. Soluble CD36 (sCD36) was determined by an in-house ELISA method as previously described [15].

### Measurement of carotid intima-media thickness

In brief, images were obtained by manual measurement using an ultrasound system with a 14 MHz linear transducer (Preirus Hi-vision Hitachi, Tokyo, Japan) with the subjects in the supine position, neck mildly extended and the head rotated contra-laterally to the side. Ultrasonographic measurements were performed by a radiologist blinded to the subject’s clinical information. To measure carotid IMT, three 10 mm segments of intima media were scanned longitudinally: the distal portion of the common carotid artery (CCA), the carotid bifurcation (BIF) and the proximal portion of the internal carotid artery (ICA). IMT was measured at the posterior (far) walls of the left carotid artery as the distance between the luminal-intimal interface and the medial-adventitial interface as described by de Groot et al. [16]. If there was evidence of carotid plaque, care was taken not to include the plaque in the IMT measurement. Plaque was defined according to The Mannheim CIMT Consensus Report [17]. IMT values were calculated as means of the three measured values.

### Determination of Achilles tendon xanthomas

Achilles tendons (AT) were examined using the linear-array transducer described above and with the subjects lying in prone position with ankles extended beyond the examination bed and feet at 90° flexion. Special attention was paid to holding the probe perpendicular to the tendon. Measurements of AT thickness (average of both tendons) were made from longitudinal scans at the point of maximum thickness. In uniformly sized tendons, measurements were taken 20 mm proximally to insertion on Calcaneus. The size of the tendon was considered normal when the maximum thickness was less than 5.8 mm [18].

### Determination of monocyte subpopulations and their CD36 expression

To avoid influence of platelet count in regard to CD36 measurements [19], we performed flow cytometry analysis on monocytes isolated from EDTA blood using the Dynabeads FlowComp Human CD14 kit and a magnetic cell sorter (Invitrogen, Carlsbad, CA, USA) following the manufacturer’s instructions. Monocytes used for gene expression analysis were sedimented by centrifugation (350 x g, 10 min.) and stored at minus 80°C in RNAlater solution (Life Technologies, Carlsbad, CA, USA). Flow cytometry on isolated monocytes was performed using a FACSCalibur flow cytometer running Cell Quest software (BD Biosciences). Subsets of classical (CD14++CD16-), intermediate (CD14++CD16+) and non-classical (CD14+CD16++) monocytes were defined according to the surface expression pattern of the lipopolysaccharide receptor CD14 and the Fcγ-III receptor CD16 according to the recommendations of the Nomenclature Committee of the International Union of Immunological Societies [20]. Absolute CD36 surface protein expression on each monocyte subset was quantified by the mean fluorescence intensity (MFI) using a mixture of PE-Quantibrite beads (BD Biosciences).

In brief, monocytes (~150.000 cells/100 μL) were incubated in the dark (4°C) for 30 min with the following monoclonal antibodies obtained from Biolegend (San Diego, CA, USA): 3 μL peridinin chlorophyll protein complex with cyanin- 5.5 (PerCP-Cy5.5)-conjugated anti-human CD14 (400 μg mL^-1^ IgG1, κ (clone HCD14), 5 μL Alexa Fluor 488-conjugated anti-human CD16 (200 μg mL^-1^ IgG1, κ (clone 3G8) and 5 μL Phycoerythrin (PE)-conjugated anti-human CD36 (6.25 μg mL^-1^ IgG2a, κ (clone 5–271). Following incubation, 250 μL of staining buffer (PBS + 2% FBS + 0.1% Sodium Azide) was added and samples were kept on ice until analysis. The optimal antibody concentrations were determined experimentally by titration experiments and concentration-matched isotype mAbs were used as controls.

A standard curve relating PE fluorescence intensity to PE surface density was then generated using PE-Quantibrite Beads tagged with 4 defined amounts of PE ranging from 200 to 70 000 molecules per bead. Using Flow Jo software (v. 8.8.7, Tree Star, Inc., Oregon, USA) the number of bound CD36 antibody molecules on each monocyte was calculated from the standard curve using the monocyte CD36 MFI, knowing that the fluorochrome-to-protein ratio of the CD36-PE conjugate is constant for each vial in a given lot of reagent and under the assumption that the stoichiometry of anti-CD36 binding to CD36 is 1:1.

### Flow cytometric measurements of monocyte-derived microparticles

Plasma levels of monocyte-derived microparticles (MMPs) were analyzed by a recently reported method using a FACSAria III digital flow cytometer [21]. Blood samples for MMP preparation were collected into sodium citrate anticoagulant at a 3.2% (0.105 M) final concentration and processed within 1 hour at room temperature. Platelet-free plasma (PFP) was prepared by a three-step centrifugation procedure and stored at -80°C until analysis. For each analysis, 50-μL of freshly thawed PFP was transferred to a TruCount tube (BD Biosciences, New Jersey, USA) containing a lyophilized pellet which releases a known number of fluorescent beads used for microparticle quantification according to the manufacturer’s instructions. Subsequently, microparticles were labeled by adding 10 μL fluorescein isothiocyanate (FITC)-conjugated Lactadherin (83 μg mL^-1^, Haematologic Technologies Inc, Vermont, USA) [22,23]. To identify MMPs, 5 μL peridinin chlorophyll protein complex with cyanin- 5.5 (PerCP-Cy5.5)-conjugated anti-human CD14 (400 μg mL^-1^ IgG1, κ (clone HCD14, Biolegend, San Diego, CA, USA)) was added immediately after Lactadherin-FITC labeling. After 30 min. of incubation (4°C, in the dark), 250 μL 0.22 μm filtered PBS was added to the labeled sample. The optimal antibody concentration was determined experimentally by titration experiments and concentration-matched isotype mAbs were used as controls.

A microparticle gate was established by preliminary standardization experiments using a blend of size-calibrated fluorescent beads with sizes ranging from 0.2 to 0.9 μm as described in [21]. The upper and the outer limits of the microparticle gate were established just above the size distribution of 0.9-μm-sized beads in a forward (FSC-A) and side scatter (SSC-A) setting (log scale) using the “auto-gate” function inside the Flow Jo software. The noise threshold of the instrument was set as the lower limit of the MP gate.

### Quantitative real-time RT-PCR

RNA was extracted from isolated monocytes using the RNeasy Micro Kit (Qiagen, Hilden, Germany). Subsequently, 4 μL of undiluted RNA solution was mixed with 10 μL MgCl_2_ 25 mM, (Roche A/S, Hvidovre, Denmark), 4 μL 10 × PCR buffer II (Roche A/S), 2 μL Oligo d(T)_16_ 50 μM (DNA Technology A/S, Aarhus, Denmark), 16 μL dNTPmix (dATP/dTTP/dCTP/dGTP) (Pharmacia Biotech, Hilleroed, Denmark), 2 μL MuLV (Moloney murine Leukaemia Virus) reverse transcriptase, (50 U/ml) (PerkinElmer Denmark A/S, Hvidovre, Denmark) and 2 μL RNase inhibitor, (20 U/ml) (PerkinElmer Denmark A/S) for a final volume of 40 μL. cDNA was synthesized by incubation at 42°C for 30 min and the process was stopped by increasing incubation temperature to 99°C for 5 min. cDNA was either used immediately or stored at -20°C. Quantitative Real-time RT-PCR was performed using the Lightcycler 480 instrument (Roche Diagnostics, Penzberg, Germany). The mRNA expression of 9 individual genes involved in cholesterol uptake (CD36), reverse cholesterol transport (ABCA1), monocyte migration (CCR2, CCR5, CX_3_CR1), inflammation (TNF-α), regulatory pathways (PPAR-γ, NFκB) as well as CD16 as an inflammatory marker were measured using the primers listed in Table 1. mRNA expression levels were normalized to the expression of [beta]_2_M ([beta]_2_-microglobulin). Primers were selected to span two exons in order to prevent amplification of genomic DNA.

**Table 1:** Characteristics of primers used for RT-PCR.

Gene Target	Primer	Sequence	Annealing temperature (°C)	Fragment length (bp)
CD36	Forward	5´-GCT GTG GCA GCT GCA TCC CAT-3´	65	318
	Reverse	5´-AAT CAT GTC GCA GTG ACT TTC CCA A-3´		
CD16	Forward	5´-GGC TGT GGT GTT CCT GGA GCC-3´	65	282
	Reverse	5´-TTG AAC ACC CAC CGA GGG GC-3´		
CCR2	Forward	5´-AGC CTG ACA TAC CAG GAC TGC CT-3´	65	228
	Reverse	5´-ACA CCA GCG AGT AGA GCG GAG G-3´		
CCR5	Forward	5´-GGC CAG AAG AGC TGA GAC ATC CG-3´	64	153
	Reverse	5´-AGG CGG GCT GCG ATT TGC TT-3´		
CX_3_CR1	Forward	5´-TGG GCT TCT TGC TGT TGG TGA GG-3´	64	220
	Reverse	5´-GGT TCC CTT GGC AGT CCA CGC-3´		
ABCA1	Forward	5´-GCC CGG CGG TTC TTG TGG AA-3´	64	220
	Reverse	5´-GGT CCG GGT TGG ACC CTG CT-3´		
NFkB	Forward	5´-ACT CGC CAC CCG GCT TCA GA-3´	64	133
	Reverse	5´-GGC AGT GCC ATC TGT GGT TGA A-3´		
PPAR-γ	Forward	5´-GCC TTG CAG TGG GGA TGT CTC A-3´	64	198
	Reverse	5´-TCG CCC TCG CCT TTG CTT TGG-3´		
TNF-α	Forward	5´-CGC TCT TCT GCC TGC TGC ACT-3´	65	196
	Reverse	5´-GCA TTG GCC CGG CGG TTC AG-3´		
[beta]_2_M	Forward	5´-AGT GGG ATC GAG ACA TGT AAG CAG-3´	64	84
	Reverse	5´-GCT ACC TGT GGA GCA ACC TGC-3´		

Primer sequence, annealing temperature and fragment length of selected target genes. Final concentration of primers was 5 pmol/μL. Abbreviations: CD36, Cluster of Differentiation 36; CD16, Cluster of differentiation 16; CCR2, C-C chemokine receptor type 2; CCR5, C-C chemokine receptor type 5; CX_3_CR1, CX_3_C chemokine receptor 1; ABCA1, ATP-binding cassette transporter 1; NFkB, Nuclear Factor-KappaB; PPAR-γ, Peroxisome proliferator-activated receptor gamma; TNF-α; Tumor necrosis factor-alpha; [beta]_2_M, [beta]_2_M, [beta]_2_-microglobulin.

A PCR mix was made on the basis of the prescription from the supplier. In brief, 3 μL sterile water, 5 μL Master Mix, (Roche Diagnostics, Penzberg, Germany), 0.5 μL sense and 0.5 μL antisense primers and 1 μL SYBRgreen (Roche Diagnostics, Penzberg, Germany) were mixed before adding 1 μL target cDNA for a total volume of 10 μL. RNA extracted from a bladder cancer cell line, HCV29 (ATCC, Manassas, Virginia, USA), was used as a calibrator control for PPAR-γ and NFκB whereas monocyte-derived RNA extracted buffy coats from healthy donors, using the EasySep Human CD14 Positive Selection Kit (STEMCELL Technologies SARL, Grenoble, France), was used as calibrator for CD36, CD16, TNF-α, CCR2, CCR5 and CX_3_CR1. The monocyte calibrator control was kindly provided by M.Sc. Aisha Rafique, Department of Clinical Biochemistry, Aarhus University. The PCR reactions were run by an initial denaturation step at 95°C for 30 s, followed by 50 cycles with a 95°C denaturation followed by annealing (temperatures are given in Table 1) for 5 s and 72°C extension for 10 s. A standard melting curve was used to check the quality of amplification. A calibration curve and positive and negative controls were included in each run. Each calibration curve was composed of serial dilutions of a RNA pool from the appropriate calibrator cell lines. As a negative control for both calibration curves, water was added instead of RNA. All PCR reactions were performed in duplicates on the same plate. Gel electrophoresis and DNA sequencing verified the specificity of each amplified RT-PCR product.

NormFinder software, an algorithm for identifying the optimal normalization gene among a set of candidates, was used to identify the most stable reference gene [24]. Comparing stability of the household candidate genes β-actin (Beta-actin), GAPDH (glyceraldehyde-3- phosphate dehydrogenase), HMBS (Hydroxymethylbilane synthase) and [beta]_2_M ([beta]_2_-microglobulin), respectively, in all 53 participating subjects resulted in the selection of [beta]_2_M as the most stable household gene.

### Statistics

Statistical analyses were carried out using the STATA 11.2 statistical program (StataCorp LP, Texas, USA). The Shapiro-Wilk’s W test was used to test the assumption of normality. Normal and non-normal distributed parameters were compared using two-tailed Student´s t-test and Mann Whitney U-test, respectively. Statistical correlation was analyzed using Spearman's rank correlation for non-normal distributed data. Predictors of IMT and MMP numbers, respectively, were identified by stepwise linear regression (backward selection) using a significance level of 10%. Variables were log transformed prior to regression. P values are two-sided and considered significant when <0.05.

## Results

### Characterization of study participants

Table 2 shows the clinical characteristics of the study groups. Routine laboratory measurements were all within age- and gender-specific reference intervals and no significant differences were observed between groups except for increased levels of total cholesterol, LDL-C, ApoB and oxLDL-C in FH compared to control subjects as well as in FH ATX+ compared to FH ATX- subjects. HDL-C levels were lower in FH subjects compared to controls, but comparable in the two FH study groups. Plasma levels of the inflammatory markers IL-6, hs-CRP, MMP-9, ICAM-1 and sCD36 as a potential marker of plaque instability were not affected by FH, except for IL-6 which was significantly increased (p = 0.022). The presence of ATX did not affect plasma levels of any of these biomarkers (Table 2).

**Table 2:** Characteristics of study participants.

Characteristics	Controls (n = 23)	FH (n = 30)	p-value^a^	FH ATX-(n = 14)	FH ATX+ (n = 16)	*p*-value^b^
Men/women	8/15	12/18	NS	7/7	5/11	NS
Age (years)	47.0 ±10.1	45.5 ±9.1	NS	43.1 ±10.2	47.5 ±7.8	NS
BMI (kg/m^2^)	23.7 ±3.3	25.5 ±5.2	NS	25.1 ±4.2	25.9 ±5.9	NS
Systolic bp (mm Hg)	120.2 ±10.8	124.9 ±14.3	NS	122.4 ±13.7	127.1 ±14.8	NS
Diastolic bp (mm Hg)	74.6 ±6.7	77.4 ±6.5	NS	76.0 ±5.5	78.7 ±7.1	NS
Platelets (10^9^/L)	239.8 ±50.7	248.6 ±60.1	NS	247 ±55.7	250 ± 65.4	NS
Leukocytes (10^9^/L)	5.1 ± 1.2	5.4 ±1.9	NS	5.0 ±1.6	5.7 ±2.2	NS
Monocytes (10^9^/L)	0.4 ±0.1	0.5 ±0.1	NS	0.4 ±0.1	0.5 ±0.2	NS
Hemoglobin (mmol/L)	8.7 ±0.6	9.0 ±0.8	NS	9.1 ±1.0	8.9 ±0.7	NS
Total-C (mmol/L)	5.1 ±0.7	9.1 ±1.8	<0.0001	8.2 ±1.1	9.9 ±2.0	<0.010
LDL-C (mmol/L)	2.9 ±0.5	7.0 ±1.8	<0.0001	6.0 ±1.1	7.9 ±1.8	0.003
HDL-C (mmol/L)	1.7 ±0.4	1.5 ±0.4	0.014	1.5 ±0.5	1.4 ±0.4	NS
oxLDL-C (Units/L)	50.0 ±11.8	100.3 ± 24.2	<0.0001	83.7 ±13.6	114.8 ±22.1	0.0001
ApoB (g/L)	0.8 ±0.1	1.7 ±0.4	<0.0001	1.4 ±0.3	1.8 ±0.3	0.002
Glucose (mmol/L)	5.4 ±0.5	5.5 ±0.4	NS	5.5 ±0.4	5.5 ±0.4	NS
ALT (U/L)	19.0 (14.0–21.0)	19.0 (17.0–34.0)	NS	20.5 (18–32)	18.5 (15.0–36.5)	NS
TG (mmol/L)	1.0 (0.8–1.2)	1.1 (0.8–1.8)	NS	1.3 (0.7–1.8)	1.1 (1.0–1.6)	NS
IL-6 (pg/ml)	0.6 (0.0–0.7)	0.8 (0.3–1.6)	0.022	0.7 (0.3–1.0)	1.0 (0.3–2.0)	NS
hs-CRP (μg/ml)	1.0 (0.8–1.9)	1.5 (0.6–4.2)	NS	1.3 (0.4–3.9)	1.9 (0.7–5.2)	NS
sCD36 (AU)	0.4 (0.3–0.5)	0.4 (0.3–0.5)	NS	0.4 (0.2–0.5)	0.4 (0.3–0.6)	NS
MMP-9 (pg/ml)	0.9 (0.6–1.2)	1.0 (0.7–2.1)	NS	1.0 (0.7–1.8)	1.1 (0.7–2.6)	NS
ICAM-1 (pg/ml)	1.1 (1.0–1.5)	1.3 (1.1–1.5)	NS	1.3 ±0.2	1.4 ±0.5	NS

Normal and non-normally distributions are shown as the mean ±standard deviation and as the median (interquartile range), respectively.

^a^Controls vs. FH subjects;

^b^FH subjects without ATX vs. FH subjects with ATX.

Abbreviations: BMI, Body mass index; ALT, Alanin-aminotransferase, TG, Triglycerides; ApoB, Apolipoprotein B; Total-C, Total cholesterol; LDL-C, LDL Cholesterol; HDL-C, HDL Cholesterol; oxLDL-C, oxidized LDL-C; bp, blood pressure; hs-CRP, High Sensitivity C-reactive Protein; IL-6, Interleukin 6; sCD36; soluble CD36, MMP-9, Matrix metalloproteinase 9; ICAM-1, Intercellular Adhesion Molecule 1; AU, arbitrary units; NS, non-significant.

### AT thickness and oxLDL-C

Mean AT thickness was increased in FH compared to control subjects (6.19 ± 1.92 mm vs. 4.78 ± 0.62 mm, p = 0.001). Absence or presence of ATX was confirmed and accordingly AT thickness was higher in the defined FH ATX+ group compared to the FH ATX- group (7.28 ± 2.08 mm vs. 4.95 ±0.35 mm, p = 0.0003). Notably, no gender- or age-related differences in AT thickness were observed within the groups. AT thickness correlated with oxLDL-C in FH subjects (Rho = 0.58, p = 0.0008) but not in controls. Likewise, AT thickness and IMT correlated in the FH group only (Rho = 0.57, p = 0.001).

### Association between intima media thickness and oxLDL-C

Mean carotid IMT was increased in FH subjects compared with the control group (0.64 ±0.12 mm vs. 0.58 ±0.07 mm, p = 0.033) and in FH ATX+ compared to FH ATX- subjects (0.69 ±0.10 mm vs. 0.57 ±0.10 mm, p = 0.002). IMT was not affected by age or gender in either group. Assuming that the impact of oxLDL-C on IMT is independent of FH, we observed a significant correlation between oxLDL-C and IMT (Rho = 0.27, p = 0.045) within the study population.

### Monocyte count and monocyte subpopulations

**Fig 1:**
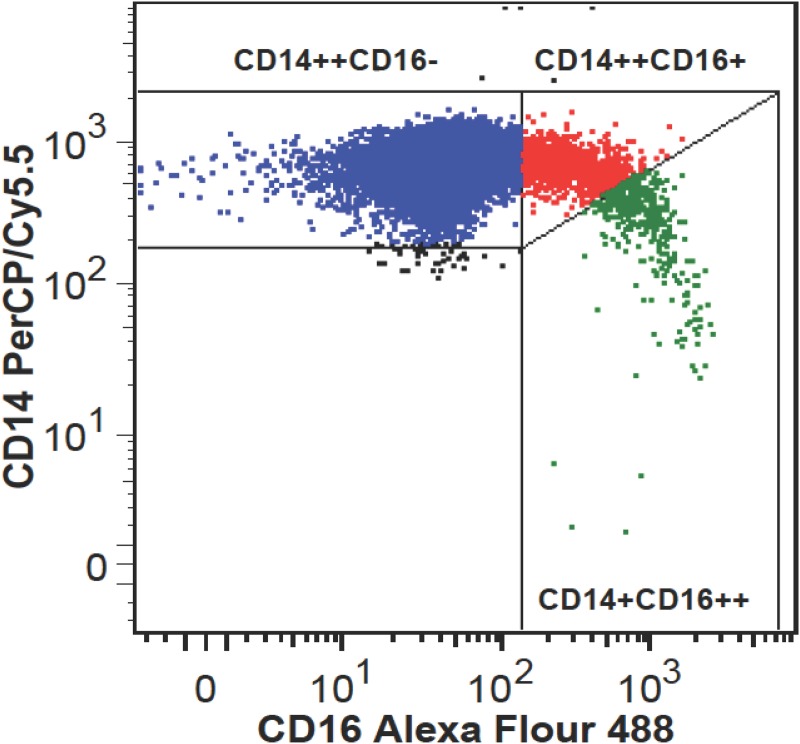
Flow cytometric analysis of monocyte subpopulations. Representative example of monocyte subsets depicted as classical CD14++CD16- monocytes (blue color), intermediate CD14++CD16+ monocytes (red color), and nonclassical CD14+CD16++ monocytes (green color).

Monocyte counts were significantly associated to IMT in all study subjects (Rho = 0.32, p = 0.019), as well in FH (Rho = 0.40, p = 0.028) and FH ATX+ (Rho = 0.52, p = 0.037) subjects when considered separately. Monocyte subpopulations were heterogeneous in regard to CD16 expression (Fig 1) and unrelated to IMT.

The percentage of intermediate monocytes was higher in FH subjects compared to controls ((2.28% (1.34–3.12) vs. (1.39% (1.02–2.38), p = 0.034) whereas no significant difference between FH ATX- and FH ATX+ subjects was found ((2.33% (1.32–2.82) vs. (2.28% (1.61–3.26), p = 0.739). Likewise, the percentage of non-classical monocytes tended to be higher in FH subjects compared to controls ((0.58% (0.40–0.98)) vs. (0.42% (0.29–0.74), p = 0.061) whereas no difference between FH ATX+ and FH ATX- subjects was found ((0.52% (0.39–1.10) vs. 0.72% (0.4–0.98), p = 0.884). Assuming that the impact of oxLDL-C on monocyte differentiation is independent of FH, it was found that the percentage of intermediate but not of non-classical monocytes correlated with plasma oxLDL-C (Rho = 0.34, p = 0.014, N = 53).

### CD36 surface expression within monocyte subpopulations

**Fig 2:**
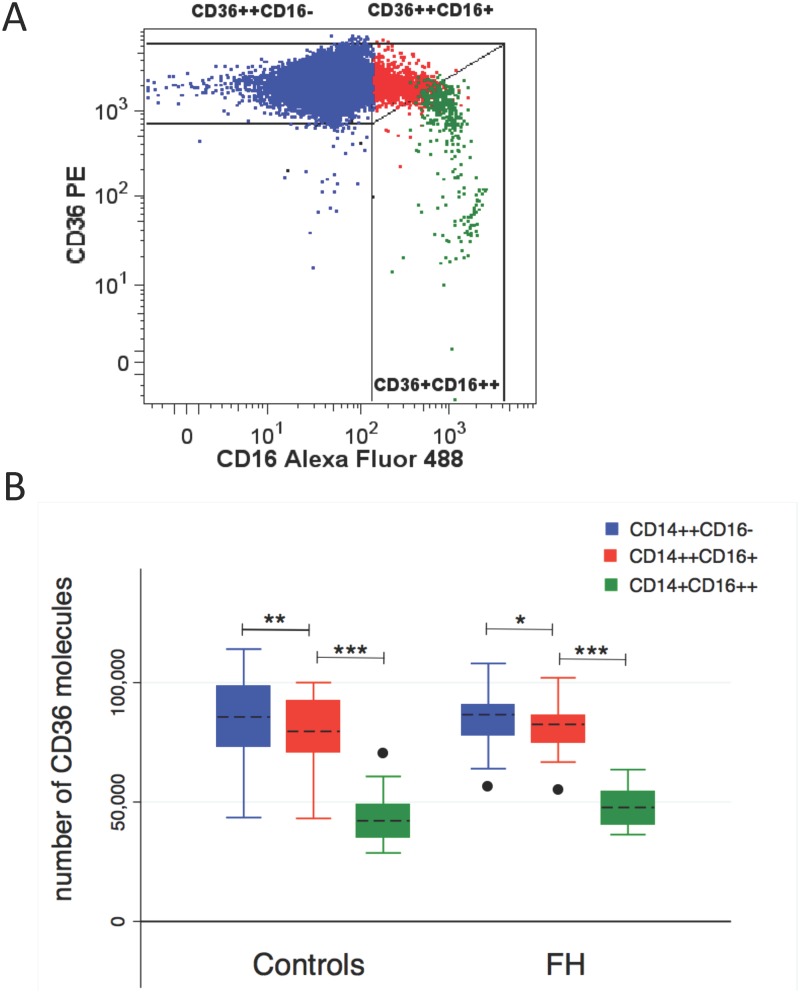
CD36 expression on monocyte subpopulations. (A) Representative flow cytometric plot of CD36 expression on monocyte subpopulations. (B) Box plot analysis of CD36 surface expression levels on classical (blue color), intermediate (red color) and nonclassical (green color) monocytes in healthy controls and FH subjects. Median (horizontal dotted line), 25% and 75% quartiles (box), 10% and 90% quartiles (whiskers). Filled circles represent outliers. *p<0.010; **p<0.005; ***p<0.0001.

In general, CD36 surface expression was demonstrated on all monocyte subpopulations. A representative flow cytometric plot of CD36 expression on monocyte subpopulations is shown in Fig 2A. Non-classical monocyte CD36 expression was elevated in FH compared to control subjects (48026 ±8007 vs. 43149 ±10091, p = 0.029, Fig 2B) whereas CD36 expression was unaffected by FH in the remaining subpopulations. The presence of ATX did not affect CD36 expression in any of the monocyte subpopulations and CD36 expression showed no correlation to oxLDL-C levels. However, CD36 expression differed among monocyte subpopulations in both FH and healthy controls, revealing a significantly reduced CD36 expression on both of the two CD16-positive monocyte subpopulations compared to the classical CD16-negative monocytes (Fig 2B).

### Expression of pro-atherogenic and pro-inflammatory genes in FH monocytes

**Fig 3:**
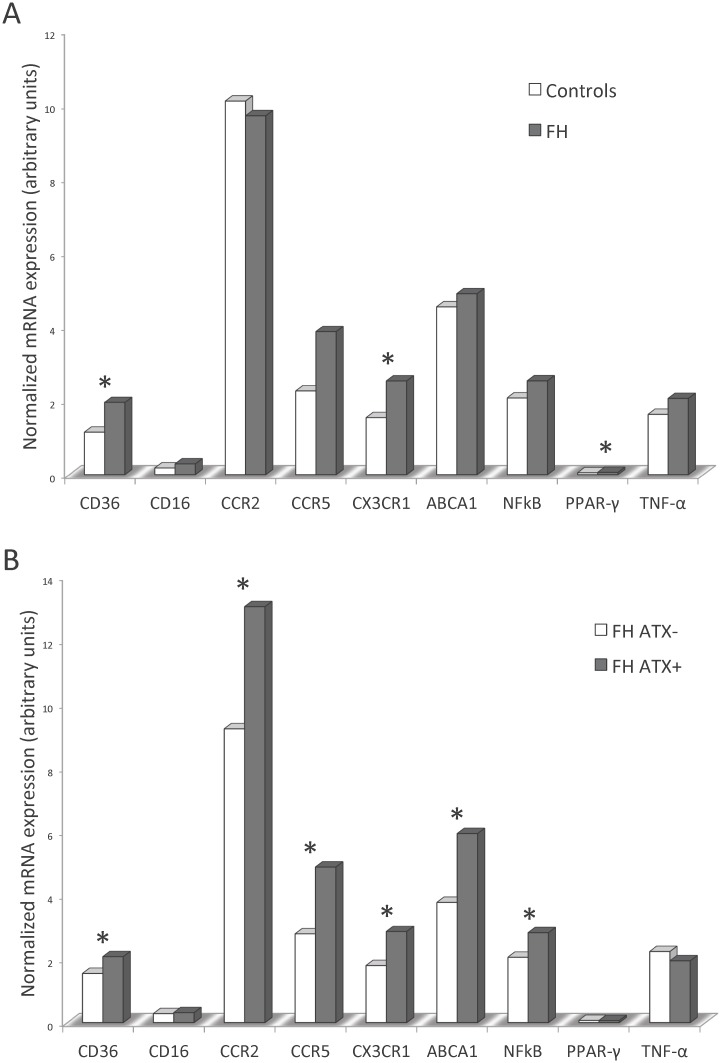
Real-time quantitative RT-PCR analysis on isolated monocytes. Expression of pro-atherogenic and pro-inflammatory genes in FH and control subjects (A) and in FH subjects with and without ATX (B). Real-time quantitative RT-PCR was performed on total RNA obtained from freshly isolated monocytes. The amount of mRNA for the gene of interest was normalized relative to [beta]_2_M mRNA, and the graphs were generated with the relative median values obtained after normalization. Mann-Whitney U test was used to compare the transcriptional expression of genes and statistically significant difference (p<0.05) is indicated by *.

The putative proinflammatory and proatherogenic phenotype of circulating monocytes was investigated at the transcriptional level by quantitative real-time RT-PCR. As illustrated in Fig 3A, FH subjects had significantly increased mRNA expression of CD36 (p = 0.008), CX_3_CR1 (p = 0.031) and PPAR-γ (p = 0.016). Moreover, FH ATX+ subjects had increased expression of CD36 (p = 0.046), CCR2 (p = 0.042), CCR5 (p = 0.018), CX_3_CR1 (p = 0.028), ABCA1 (p = 0.009) and NFκB (p = 0.018) when compared to FH ATX- subjects (Fig 3B).

CD36 mRNA and protein levels were unrelated. Correlation analysis was applied in order to test the putative association of oxLDL-C with monocyte expression of genes involved in the atherosclerotic process. Within the entire study population, as well as in FH subjects, oxLDL-C was positively correlated with the expression of CD36, CD16, CCR2, CCR5, CX_3_CR1 and NFκB but not PPAR-γ, TNF-α or ABCA1 (Table 3).

**Table 3:** Correlation analysis of oxLDL-C and expression levels of genes involved in the atherosclerotic process.

	oxLDL-C			
*mRNA*	All		FH	
	Rho	p-value	Rho	p-value
CD36	0.39	0.004	0.44	0.015
CD16	0.27	0.049	0.51	0.004
CCR2	0.33	0.017	0.40	0.030
CCR5	0.32	0.021	0.48	0.008
CX_3_CR1	0.37	0.007	0.45	0.013
PPAR-γ	0.20	NS	-0.11	NS
NFkB	0.29	0.032	0.46	0.010
TNF-α	0.25	NS	0.18	NS
ABCA1	0.22	NS	0.33	NS

mRNA levels are normalized to the expression levels of the housekeeping gene [beta]_2_M and given in arbitrary units. NS; non significant. A p-value≤0.05 was considered statistically significant.

### Possible contributors to IMT

The relationship between IMT and the expression levels of the pro-atherogenic and pro-inflammatory genes in monocytes was investigated. Within the entire study population IMT correlated positively with the expression of CD36, CCR2, CCR5, CX_3_CR1, NFκB and TNF-α but not with PPAR-γ, CD16 or ABCA1. In FH subjects, IMT correlated significantly with the expression of CCR2, CCR5, CX_3_CR1 and NFκB and approached significance with CD36 (p = 0.053) whereas no correlation with CD16, PPAR-γ, TNF-α or ABCA1 was present (Table 4).

**Table 4:** Correlation analysis of IMT and expression levels of genes involved in the atherosclerotic process.

	IMT			
*mRNA*	All		FH	
	Rho	p-value	Rho	p-value
CD36	0.40	0.003	0.36	0.053
CD16	0.26	NS	0.14	NS
CCR2	0.41	0.003	0.39	0.031
CCR5	0.44	0.001	0.40	0.027
CX_3_CR1	0.49	0.0002	0.42	0.022
PPAR-γ	0.26	NS	0.08	NS
NFkB	0.47	0.0004	0.36	0.047
TNF-α	0.29	0.039	0.15	NS
ABCA1	0.14	NS	0.23	NS

mRNA levels are normalized to the expression levels of the housekeeping gene [beta]_2_M and given in arbitrary units. NS; non significant. A p-value≤0.05 was considered statistically significant.

In order to study components involved in the initial recruitment of circulating monocytes into the vessel wall, induction of inflammation and accumulation of cholesterol at the transcriptional level, a statistical model was tested in which IMT was the dependent variable and variables showing correlation to IMT in healthy and FH subjects (Table 4) were included as independent variables. Stepwise linear regression analysis identified CX_3_CR1 as the sole contributor to IMT within the entire study population (R^2^ = 0.30; p<0.0001) as well as in FH subjects only (R^2^ = 0.23; p = 0.0008).

### Indication of MMP release from proinflammatory monocytes

Total MMP numbers were higher in FH subjects (10 (7–14) MMPs / μL plasma) compared with controls (5 (3–11) MMPs / μL plasma), p = 0.004 and unaffected by the presence of ATX. The hypothesis that cholesterol fractions, particularly oxLDL-C, promote the release of MMPs from proinflammatory monocytes was tested. Considering that the impact of cholesterol fractions on MMP release is independent of FH, correlation analysis revealed a positive correlation between MMP numbers and LDL-C (Rho = 0.41; p = 0.003) and oxLDL-C (Rho = 0.35; p = 0.010) whereas HDL-C correlated negatively (Rho = -0.34; p = 0.014). MMP numbers and monocyte counts were not correlated. Within the entire study population, MMP numbers correlated with the percentage of both intermediate (Rho = 0.37, p = 0.007) and non-classical monocytes (Rho = 0.34, p = 0.012) whereas in FH subjects, only the correlation with the intermediate monocytes remained significant (Rho = 0.45, p = 0.013).

The possible significance of proinflammatory monocytes and lipoprotein-associated oxidative stress for circulating MMP numbers was investigated using stepwise linear regression analysis. Gender was included as a variable as it is known to affect MP levels [21]. Regression analysis identified oxLDL-C, intermediate monocyte percentage and gender as independent predictors of MMP levels, both within the entire study population (R^2^ = 0.37; p<0.0001) and in FH ATX+ subjects (R^2^ = 0.59; p = 0.011) (Table 5).

**Table 5:** Stepwise linear regression analysis (backward selection) to study the independent variables predicting MMPs in FH subjects.

	All		FH		FH ATX+	
	R^2^ = 0.37, p<0.0001		R^2^ = 0.24, p = 0.024		R^2^ = 0.59, p = 0.011	
Independent variables	β	p-value	β	p-value	β	p-value
oxLDL-C	0.34	0.007	-	-	0.42	0.082
Intermediate	0.23	0.025	0.38	0.025	0.38	0.087
Non-classical	-	-	-	-	-	-
Gender	0.49	0.019	0.44	0.023	0.70	0.004

The following independent variables were considered for the model: oxLDL-C (U/L), intermediate monocytes (percentage), non-classical monocytes (percentage) and gender. β = standardized regression coefficient. A p-value≤0.1 was considered statistically significant.

## Discussion

The results presented herein suggest an association between the clinical manifestations of FH and oxLDL-C, a well-recognized marker of lipoprotein-associated oxidative stress and oxidative stress in the arterial wall [25]. We report that IMT is related to AT thickness in FH and to the transcriptional level of genes related to an oxLDL-C-induced response and chemokine receptor expression in monocytes, and that circulating MMPs are associated with the percentage of intermediate monocytes and plasma oxLDL-C.

### Relationship between lipoprotein-associated oxidative stress, AT thickness and subclinical atherosclerosis

IMT is a well-established measure of subclinical atherosclerosis [16]. Like previous studies, we demonstrate increased IMT in FH, particularly in the presence of ATX [11,26]. The formation of ATX has been proposed to involve uptake of oxLDL-C by macrophages within the tendons [9]. Apart from a recent study showing that macrophages from FH ATX+ subjects are more sensitive to the actions of oxLDL-C compared with cells from FH ATX- subjects [27], the reason why only some FH subjects develop ATX is largely unknown. The present finding of an association between oxLDL-C and AT thickness in FH supports a key role of oxLDL-C for the development of ATX. The apparently discordant finding by Tsouli et al. [28] that autoantibodies against oxLDL-C, but not elevated plasma oxLDL-C, correlated with AT thickness in FH subjects may be related to differences in the clinical definition of ATX since our study use a well-defined AT thickness threshold value like in [18] whereas Tsouli and coworkers divided FH subjects into three groups according to the tendon echostructure grade.

The observed association between IMT values and plasma oxLDL-C is in agreement with previous publications [29,30]. Interaction between CD36 and oxLDL-C promote a pro-inflammatory and pro-atherogenic response [31] and the increased expression of pro-atherogenic and pro-inflammatory genes in FH monocytes, particularly observed in monocytes derived from FH subjects with ATX, as well as their correlation with plasma oxLDL-C and IMT demonstrated herein, supports the important contribution of oxLDL-C in the progression of atherosclerosis.

### Proinflammatory monocytes are increased in hypercholesterolemia

An association between monocyte counts, subclinical carotid atherosclerosis and plaque formation was previously reported from a study of a healthy population [32] and the association between IMT and monocyte counts; especially in FH ATX+ subjects as presented here, supports this potential influence of monocyte number on the development of macrovascular complications. A possible link between CD16-positive monocytes and hypercholesterolemia was previously suggested in early 1996 by Rothe and colleagues [33]. To the best of our knowledge, the present study is the first report of increased proportions of intermediate monocytes in FH subjects. However, a relation between subclinical atherosclerosis and any of the investigated CD16-positive subpopulations was not confirmed in our study, thus somewhat in contrast to a large cohort study by Rogacev et al. showing that intermediate monocytes were independently predicting cardiovascular events [34]. A plausible explanation for this disparity is that the design of this study used vascular insults as primary endpoint as opposed to the present study group of FH patients without any clinical symptoms of cardiovascular disease. Moreover, the flow cytometric approaches used to describe the monocyte subpopulations are not identical as Rogacev and coworkers analyzed monocytes in whole blood whereas the flow cytometric data presented herein are based on isolated monocytes. Although the precise function of intermediate monocytes in vivo remains somewhat uncertain, a possible function under hyperlipidemic conditions could be the detoxifying of oxLDL-C along the endothelial layer as proposed by Mosig and colleagues [35] or they could display a protective role by enhancing the clearance of oxLDL-C from the circulation. Finally, a higher proportion of intermediate monocytes may simply reflect a response to an inflammatory environment in FH.

### Increased expression of chemokine receptors involved in monocyte migration

Pathological mechanisms leading to plaque development have been widely studied in murine models of atherosclerosis. Results from mice studies indicate that monocyte recruitment into plaques is dependent on the chemokine receptors CCR2, CCR5 and CX_3_CR1 [5,36,37]. In vitro studies indicate that this recruitment and long-range crawling behavior on the resting endothelium is mediated by the interaction of monocyte CX_3_CR1 with CX_3_CL2 (fractalkine) locally expressed on activated endothelium [4,38,39]. The present study demonstrates an increased CX_3_CR1 expression in FH subjects, particularly in FH ATX+ subjects, supporting a possible involvement of monocyte CX_3_CR1 in subclinical atherosclerosis during pathological conditions such as hypercholesterolemia. The increased expression levels of all the investigated chemokine receptors in FH subjects with ATX suggest simultaneous roles in atherosclerosis progression as suggested earlier [5,37,39].

### CD36 surface expression on monocyte subpopulations

CD36 plays an important role in monocyte uptake of oxLDL-C leading to foam cell formation and thickness of the vessel wall [40]. A previous study reported increased CD36 surface expression on both CD16-positive and CD16-negative monocytes derived from homozygous FH subjects when compared to healthy controls [35]. In the present study of heterozygous FH subjects, monocyte CD36 surface expressions were similar among the study groups whereas at the transcriptional level we observed increased CD36 expression in FH subjects, as well as in FH ATX+ compared to FH ATX- subjects. In support of this latter finding, PPAR-γ expression which is believed to stimulate oxLDL-C-induced CD36 transcription [41] was likewise increased in FH subjects. Despite particular care regarding quantitative issues, no correlation between CD36 surface expression and CD36 mRNA levels was found. This inconsistency between CD36 mRNA and membrane protein levels may be due to the storage of preformed CD36 protein in intracellular pools and receptor recycling [42].

In line with earlier studies [43,44] we observed an overall and significantly lower CD36 surface expression on intermediate compared to classical monocyte and on non-classical compared to the intermediate monocytes. Although intermediate and non-classical monocytes express fewer surface CD36 compared to classical monocytes, these subpopulations may take up significantly more oxLDL-C as reported in [35,44,45]. Increased pressure on this mechanism like increased oxidative stress and oxLDL-C may thus induce accelerated cholesterol accumulation and local inflammation in the vessel wall.

Overall, these findings suggest that part of the background for accelerated atherosclerosis in FH, particular in the presence of ATX, may be increased lipoprotein-associated oxidative stress leading to elevated CD36 expression and proinflammatory monocytes and thus potentially accelerated cholesterol accumulation [41].

### A putative link between subclinical atherosclerosis and expression of pro-atherogenic and pro-inflammatory genes in circulating monocytes

Most of the current knowledge on the pathological mechanisms at the cellular level behind atherosclerosis development is based on in vitro studies and animal models. In the present study we have investigated some of these mechanisms in humans before the development of overt atherosclerosis. Our results from FH patients demonstrate significant relationships between IMT and monocyte expression of genes involved in cholesterol uptake (CD36), monocyte migration (CCR2, CCR5 and CX_3_CR1) and regulatory pathways (PPAR-γ, NFκB). These results support the widely acknowledged model of macrophage activation and atherosclerosis progression as reviewed in [40,46] operating in individuals without symptoms of cardiovascular disease. Moreover, we identified CX_3_CR1 as an independent predictor of IMT. Thus, the transcriptional signature found in circulating and activated monocytes may mimic that of activated macrophages within the arterial wall. Our findings suggest that some pathophysiological mechanisms in monocytes related to atherosclerosis development, as proposed by animal models and in vitro studies, may be similar in human monocytes.

### A possible link between MMPs and proinflammatory monocytes

Previous reports of increased plasma levels of MMPs in subjects with acute coronary syndrome and with high autoantibodies against oxLDL-C [47], and of an increased release of MMPs upon cholesterol enrichment of human monocyte/macrophages [48], suggest a link between lipoprotein-associated oxidative stress, monocyte/macrophage activation and MMP release and may also reflect some of the underlying mechanism involved in the atherosclerotic process. We herein report on an association between plasma MMPs, oxLDL-C and the subset of intermediate monocytes known to posses a high capacity for oxLDL-C uptake. To our knowledge, this is the first report to suggest an association between MMP number and intermediate monocytes. Our data support a model in which MMPs are released from this monocyte subpopulation upon stimulation by oxLDL-C. Release of MMPs may enhance inflammation and cause endothelial dysfunction [49,50]. Activation of monocytes is associated with vascular endothelial damage, thus high concentrations of MMPs may indicate vascular complications in FH subjects, especially those with ATX.

As the major limitation, our study cannot prove causality because of its cross-sectional design. However, by applying a number of technologies and by studying processes involved in atherosclerosis development at different levels we aimed at addressing some of the general mechanisms believed to be involved in the progression of atherosclerosis. Since diagnosis of FH was an inclusion criterion, these patients had been treated with lipid-lowering medication for ethical reasons. Although this medication was withdrawn for eight weeks before the study, this treatment may have resulted in less advanced atherosclerosis than would have been the case without treatment. As another limitation, we explored atherosclerosis-related gene expression at mRNA level only, except for CD16 and CD36, which were analyzed by flow cytometry. Hence, post-transcriptional and/or posttranslational regulation of the investigated genes cannot be excluded. RNA analysis was applied on CD14 purified monocytes and extraction of RNA from sorted monocyte subsets would have been ideal, yet technically challenging due to overlapping expression of monocyte markers. However, in future studies flow cytometric analysis of each of the investigated chemokine receptors on monocyte subsets could be investigated. Finally, a limited sample size may be the reason why markers such as inflammatory markers are elevated in FH patients with ATX compared with those without ATX, and in FH patients compared with controls without reaching statistical significance.

A major strength of our study is that it provides an extensive dataset on human monocyte heterogeneity and transcriptional signature in hypercholesterolemia before the development of atherosclerosis. Moreover, the finding of a possible link between CD16-positive intermediate monocytes, lipoprotein-associated oxidative stress (oxLDL-C) and circulating MMPs encourage further clinical studies on this topic.

## Conclusions

In this study a number of mechanisms based on animal models and in vitro studies and proposed to constitute the pathophysiological mechanisms behind the role of monocytes in atherosclerosis have been demonstrated in patients with FH. Our data suggest that lipoprotein-associated oxidative stress is involved in accelerated atherosclerosis in FH, particularly in the presence of ATX, possibly by inducing pro-inflammatory monocytes and increased release of MMPs along with elevated monocyte expression of oxLDL-C-induced and atherosclerosis-related genes.
